# The skin as a window to the gut: A case of carcinoid syndrome

**DOI:** 10.1002/ccr3.8641

**Published:** 2024-03-07

**Authors:** Catarina Alves Costa, Tânia Lopes, Ana Patrícia Rodrigues, Nuno Jorge Lamas, Célia Cruz

**Affiliations:** ^1^ Internal Medicine Department Centro Hospitalar Universitário de Santo António (CHUdSA), Porto, Largo Professor Abel Salazar Porto Portugal; ^2^ Anatomic Pathology Service, Pathology Department Centro Hospitalar Universitário de Santo António (CHUdSA), Porto, Largo Professor Abel Salazar Porto Portugal; ^3^ Life and Health Sciences Research Institute (ICVS), School of Medicine University of Minho Braga Portugal; ^4^ ICVS/3B's, PT Government Associate Laboratory University of Minho Braga Portugal

**Keywords:** carcinoid syndrome, chronic diarrhea, neuroendocrine tumors, somatostatin analogs, venous telangiectasia

## Abstract

Neuroendocrine tumors (NETs) are a group of uncommon neoplasms derived from enterochromaffin or Kulchitsky cells (that secrete serotonin or other molecules into the bloodstream), which can manifest with symptoms of hormonal overproduction, namely carcinoid syndrome (CS). This can be the presenting feature in patients with advanced disease. We report the case of a 66‐year‐old woman presenting with chronic diarrhea, facial venous telangiectasia and elevated urinary 5‐hydrocyindoleacetic acid levels. A 68‐Ga DOTATOC PET/CT scan revealed an ileal mass and lesions consistent with liver, ovary and bone metastasis. A liver biopsy confirmed the diagnosis of well‐differentiated NET G1. Therapy with somatostatin analogs achieved symptom control, but the liver disease progressed and the patient passed away after 2 years of follow‐up. The challenge of diagnosing CS resides in its heterogeneous manifestations, which may range from mild to life‐threatening conditions. In this case, the cutaneous findings of venous telangiectasia strongly pointed to the correct diagnosis. Treatment can also be difficult due to refractory symptoms and inevitable progression of disease, highlighting the importance of early detection and thorough disease staging.

## 1 INTRODUCTION

Neuroendocrine tumors (NETs) are heterogeneous neoplasms that are classified according to their grade, primary tumor site and their ability to secrete vasoactive hormones. Nonfunctional NETs have no hormone‐related clinical features in opposition to functional NETs, which have a highly variable clinical presentation based on the type and amount of hormone produced. The most frequent form of hormone overproduction is carcinoid syndrome (CS)^1^ and its symptoms are often mild and unspecific, leading to protracted diagnosis and worse prognosis.^2^ The diagnosis hinges on the clinical findings and laboratory evidence of excess production of serotonin metabolites.^3^ Somatostatin analogs (SSA) are the cornerstone of treatment, but this may be a challenge in the face of metastatic disease.^4^ This case highlights the importance of a comprehensive physical examination and complete staging in cases of CS to avoid misdiagnosis and propose personalized treatment.

## 2 CASE HISTORY/EXAMINATION

A 66‐year‐old woman with a history of smoking and uncontrolled hypertension presented to the emergency department with complaints of daily watery diarrhea for the previous 18 months. The diarrhea episodes were progressively becoming more frequent over time (up to 15 times a day, even during the night) and did not improve with fasting. She also reported anorexia, unquantified weight loss and night sweats, but denied episodes of flushing.

**FIGURE 1:**
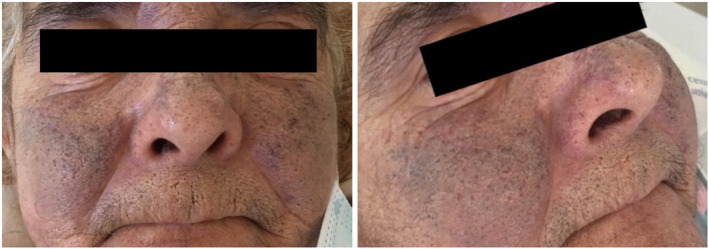
Malar and nasal venous telangiectasia.

On admission, the patient was described as dehydrated, hypertensive (BP 190/90 mmHg) and tachycardic (HR 110 bpm). A palpable painful hepatomegaly and marked confluent telangiectasias of the nose and malar regions (Figure 1) were also evident.

## 3 METHODS

Initial laboratory tests revealed elevated inflammatory markers, mild hyponatremia and hypokalemia with normal kidney function, hypoalbuminemia, direct hyperbilirubinemia, and elevated cholestatic liver enzymes. These and other laboratory data can be found on Table 1.

**TABLE 1:** Laboratory data on admission.

Variable	On Admission	Reference range
White‐cell count (/μL)	14,430	4000–11,000
Neutrophils (/μL)	11,460	2000–75,000
Hemoglobin (g/dL)	11.8	13–17
Mean corpuscular volume (fL)	81.5	83–101
Platelets (/μL)	536,000	150,000–40,000
Erythrocyte sedimentation rate (mm)	79	0–12
C Reactive protein (mg/L)	132	0–5
Creatinine (mg/dL)	0.69	0.5–0.9
Urea (mg/dL)	30	10–50
Sodium (mmol/L)	132	135–145
Potassium (mmol/L)	3.0	3.5–5
Albumin (g/dL)	2.49	3.5–5
Vitamin B12 (pg/mL)	854	191–663
Folic acid (ng/mL)	1.0	3.9–26.8
Total bilirrubin (mg/dL)	1.82	0.2–1
Direct bilirrubin (mg/dL)	1.5	0–0.3
Aspartate transaminase (U/L)	41	10–30
Alanine transaminase (U/L)	46	10–36
Alkaline phosphatase (U/L)	684	35–104
Gamma‐glutamyl transferase (U/L)	290	290
NT‐proBNP (pg/mL)	1955	0–125
Ferritin (ng/mL)	712	12.8–454
Transferrin saturation (%)	10	15–45
International normalized ratio	1.3	‐

Due to the abnormalities in the liver biochemical tests, the patient underwent abdominal ultrasonography, which confirmed hepatomegaly with multiple space‐ occupying lesions consistent with diffuse liver metastasis.

**FIGURE 2:**
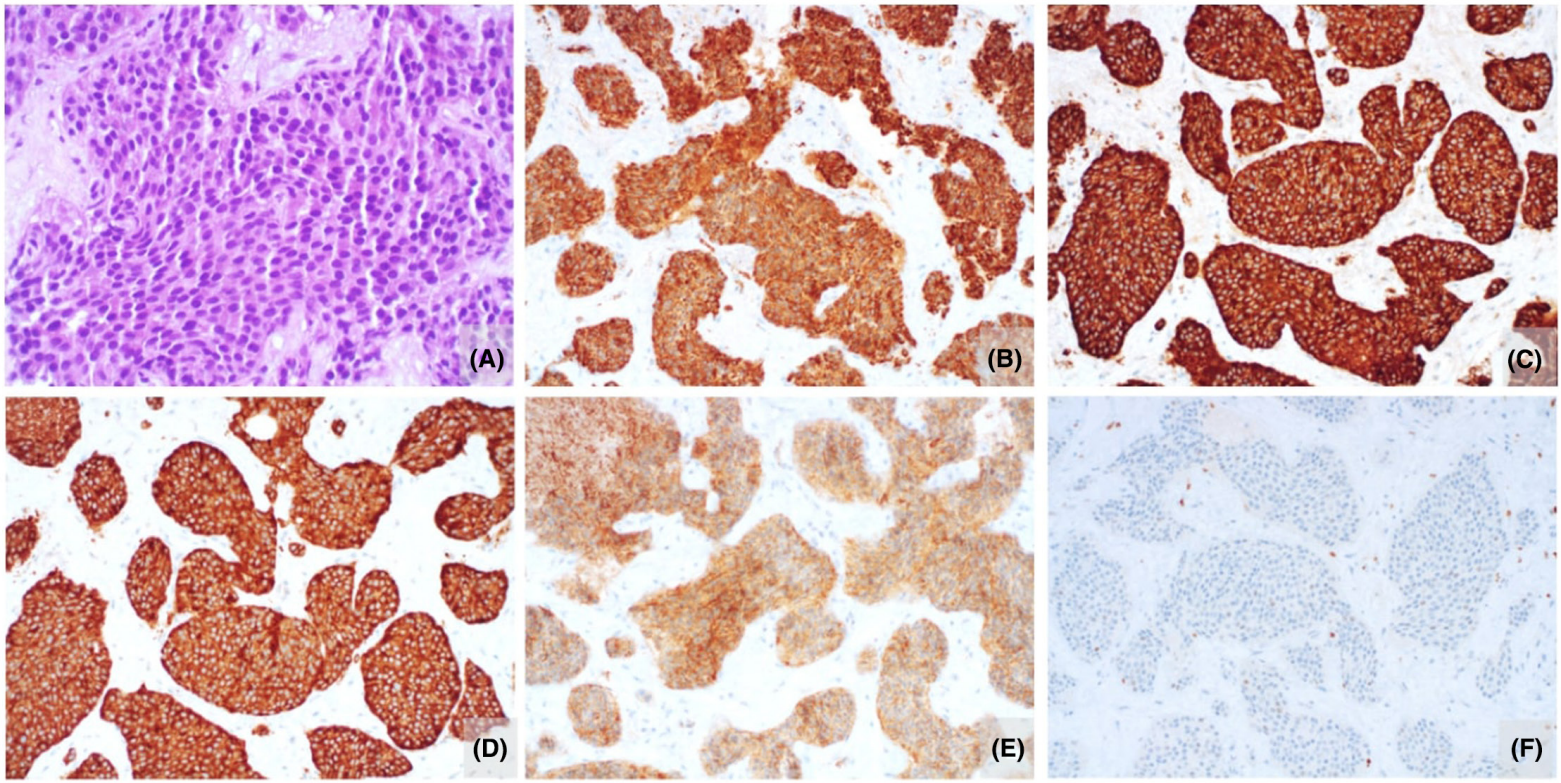
Well‐Differentiated Neuroendocrine Tumor (NET) G1: a malignant neoplasm composed of nests and trabeculae of monomorphic cells with round, hyperchromatic nuclei, without obvious nucleolus and scant eosinophilic cytoplasm was observed (A) H&E, 400× magnification). Mitotic figures were scarce. The neoplastic cell immunoreactive for CAM5.2 (B), 200× magnification), Chromogranin A (C), 200× magnification), Synaptophysin (D), 200× magnification) and CD56 (E), 200× magnification). The proliferative index (% Ki‐67) was below 1% (F), 200× magnification).

The combination of chronic diarrhea, facial telangiectasia and liver metastasis immediately raised suspicion for CS. Computed tomography scan of the chest, abdomen and pelvis was remarkable for a hypervascular intraluminal mass in the terminal ileum, hepatomegaly (207 mm) with more than 10 solid hypervascular lesions with necrotic/cystic areas, the largest measuring 108 mm, as well as bilateral ovarian metastatic nodules (Figure 2). Further laboratory tests documented high blood levels of chromogranin A (441.7 ng/mL) and elevated 24‐h urinary 5‐hydrocyindoleacetic acid (5‐HIAA) concentration (>75 mg/day). A transthoracic echocardiogram exhibited thickening and retraction of the tricuspid valve leaflets, as well as moderate to severe regurgitation, coherent with carcinoid heart disease. A liver biopsy was performed, establishing the diagnosis of well differentiated NET G1 (Figure 2).

**FIGURE 3:**
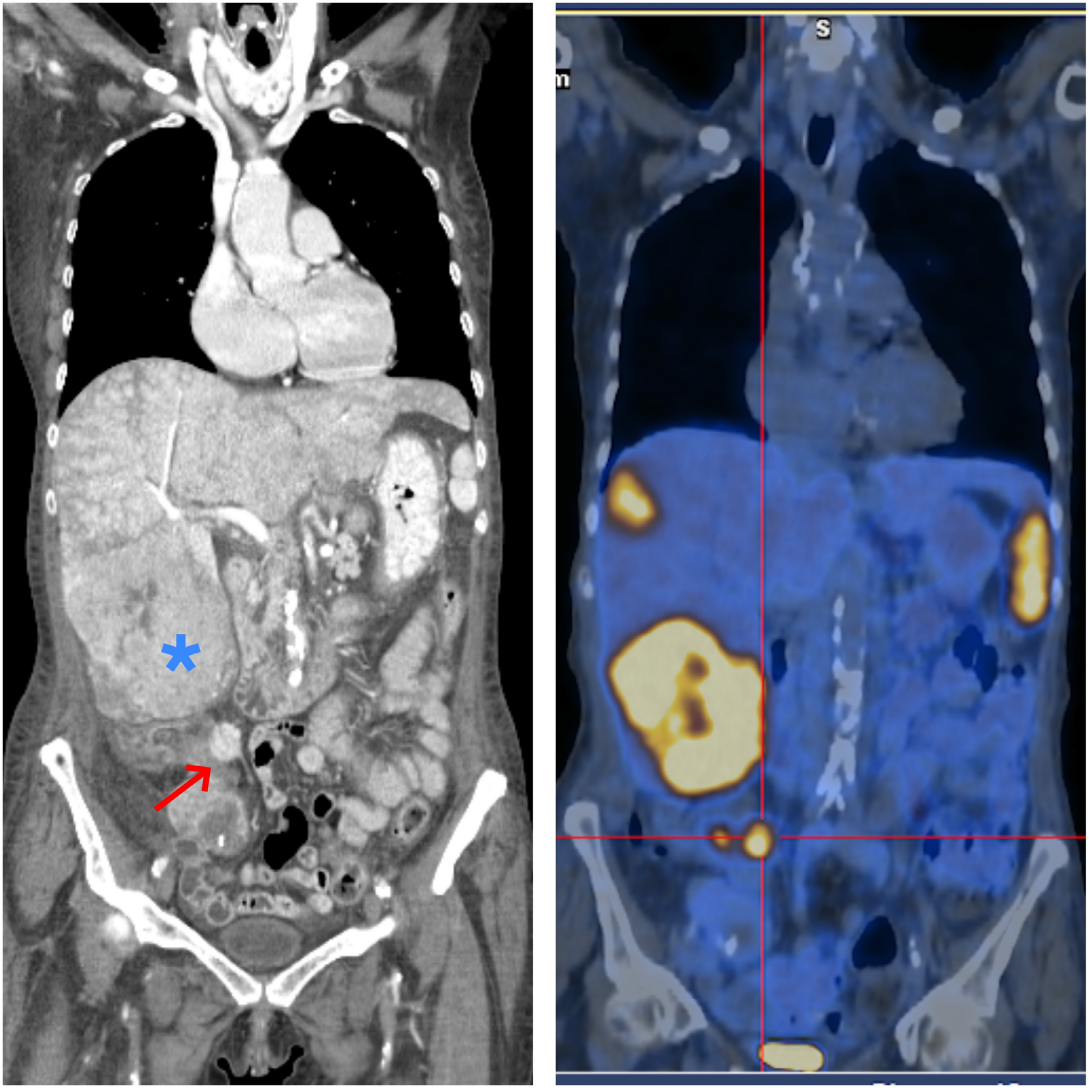
LEFT: CT scan showing a mass on the terminal ileum (red arrow) and diffuse liver metastasis (blue star). RIGHT: 68‐Ga DOTATOC PET/CT scan revealing the ileal and liver lesions as expressing somatostatin receptors.

Staging was completed with a 68‐Ga DOTATOC PET/CT scan (Figure 3), which also reavealed previously undetected vertebral metastatic focus.

## 4 CONCLUSION AND RESULTS

The patient was ultimately diagnosed with ileal NET with extensive metastatic burden and subsequent CS. She was started on ocreotide LAR with marked symptom improvement and stability of the lesions on 68‐Ga DOTATOC PET/CT scan. However, she eventually developed progression of the disease with liver failure and passed away after 2 years of follow‐up.

## 5 DISCUSSION

The true incidence of CS remains to be firmly established, but it has been reported to occur in nearly 20% of patients diagnosed with NETs.^5^ There is a strong association with tumor grade and advanced stages of the disease.^2^, ^5^ While NETs can arise anywhere in the gastrointestinal tract, midgut tumors are the most frequently associated with CS,^3^ which was the case with this patient. Other less common localizations include the lung, gonads and the pancreas.^1^, ^3^ The pathophysiology of CS is still not completely understood, but the secretion of serotonin (5‐HT) appears to be one of the hallmark mechanisms.^2^, ^3^ However, more than 40 vasoactive substances have been implicated in the development of this disorder, including histamine, kallikrein, prostaglandins, and tachykinins, among others.^6^ Even though the precise role of each mediator is uncertain, they are linked to the clinical manifestations. Patients with CS may present with flushing (90%), diarrhea (60%–80%), abdominal pain (35%), bronchospasm (15%) and pellagra (5%).^2^ Our patient lacked the classical symptom of flushing, but displayed a less common cutaneous manifestation of CS: venous telangiectasia. Telangiectasia is due to prolonged exposure to substances with vasodilator properties and is usually preceded by episodic flushing, which made this manifestation all the more surprising in this case. This unexpected finding triggered the suspicion of CS and fast‐tracked the diagnostic process.

Since 5‐HT is postulated to stimulate fibroblast growth and fibrogenesis,^3^ CS can be complicated by right heart valvulopathy and mesenteric fibrosis.^2^ Carcinoid heart disease may be asymptomatic, but portends a prognosis, and an isolated elevation in NT‐proBNP levels may herald cardiac involvement,^2^ which was the case with our patient. The tricuspid regurgitation may also have contributed to the abnormal liver panel by causing liver venous congestion.

The CS diagnosis in our case was made possible by combination of the suggestive clinical picture and elevated urinary levels of 5‐HIAA, a serotonin metabolite. The liver biopsy was instrumental to finally establish the diagnosis and characterize the NET. Imaging was important in the identification of the primary lesion and also in the NET staging. In addition, novel imaging techniques like 68‐Ga DOTATOC PET/CT scan are more sensitive than traditional imaging methods in recognizing smaller primary or secondary lesions,^7^ as showcased by this report.

SSA are considered first‐line treatment for CS and have demonstrated effectiveness in controlling symptoms and improving quality of life.^3^ Some studies have also suggested that these drugs may inhibit tumor growth.^3^ Our patient initially responded favorably with a paucity of side effects. In spite of optimized SSA therapy, in part due to possible tachyphylaxis,^8^ some individuals may develop refractory disease and require other options like telotristat, everolimus, peptide receptor radionuclide therapy, or interferon alpha.^4^ Surgical intervention is limited to patients with resectable disease. Selected patients can be candidates for liver directed therapies like embolization, radiofrequency ablation or selective internal radiotherapy, but these cytoreductive procedures need further study in CS^4^ and our patient was not considered for this therapy due to the massive liver involvement.

CS is a rare paraneoplastic syndrome usually associated with metastatic NETs that may be a diagnostic challenge due to its heterogeneous manifestations, which may range from mild to life‐threatening. Clinicians should consider a diagnosis of CS in patients presenting with skin findings due to vasodilation and chronic diarrhea. Prognosis for patients with metastatic carcinoid tumors has improved during the last decade mainly due to combining new diagnostic and treatment modalities, highlighting the importance of early detection, accurate histopathological diagnosis and thorough staging.

## AUTHOR CONTRIBUTIONS

**Catarina Alves Costa:** Conceptualization; investigation; writing – original draft. **Tânia Lopes:** Conceptualization; investigation; writing – original draft. **Ana Patrícia Rodrigues:** Visualization; writing – review and editing. **Nuno Jorge Lamas:** Visualization; writing – review and editing. **Célia Cruz:** Conceptualization; writing – review and editing.

## FUNDING INFORMATION

None.

## CONFLICT OF INTEREST STATEMENT

The authors report no conflict of interest.

## CONSENT

Written informed consent was obtained from the patient to publish this report in accordance with the journal's patient consent policy.

## Data Availability

All data regarding this study has been reported in the manuscript. Please contact the corresponding author if you are interested in any further information.
